# Oxygen Dependence of Flight Performance in Ageing *Drosophila melanogaster*

**DOI:** 10.3390/biology10040327

**Published:** 2021-04-14

**Authors:** Valeriya Privalova, Ewa Szlachcic, Łukasz Sobczyk, Natalia Szabla, Marcin Czarnoleski

**Affiliations:** Institute of Environmental Sciences, Faculty of Biology, Jagiellonian University, Gronostajowa 7, 30-387 Kraków, Poland; valeriya.privalova@doctoral.uj.edu.pl (V.P.); ewa.szlachcic@doctoral.uj.edu.pl (E.S.); lukasz.sobczyk@uj.edu.pl (Ł.S.); szabla.natalia@gmail.com (N.S.)

**Keywords:** ageing, climbing, hypoxia, insects, locomotor activity, oxygen limitation, physiological performance, senescence, wing-beat frequency, wing load

## Abstract

**Simple Summary:**

Studying insects as they age helps us better understand human ageing. Insects are excellent at delivering oxygen to flight muscles, but we do not know whether they lose their flight ability and tolerance to poor oxygen conditions with age. We studied two types of physical activity in ageing fruit flies, measuring how quickly they flap their wings and climb walls. We measured flight in either normal air or air with low oxygen availability. As we expected, young flies were better climbers than old flies, and flies flew more slowly when oxygen was low. Against expectations, young and old flies flew similarly and equally tolerated poor oxygen conditions. Overall, we suggest that insects maintain their flight abilities with age, which is surprising because insect flight requires enormous amounts of oxygen and energy. Moreover, we suggest that habitats with a poor oxygen supply (e.g., those at high elevations) can become challenging for flying insects.

**Abstract:**

Similar to humans, insects lose their physical and physiological capacities with age, which makes them a convenient study system for human ageing. Although insects have an efficient oxygen-transport system, we know little about how their flight capacity changes with age and environmental oxygen conditions. We measured two types of locomotor performance in ageing *Drosophila melanogaster* flies: the frequency of wing beats and the capacity to climb vertical surfaces. Flight performance was measured under normoxia and hypoxia. As anticipated, ageing flies showed systematic deterioration of climbing performance, and low oxygen impeded flight performance. Against predictions, flight performance did not deteriorate with age, and younger and older flies showed similar levels of tolerance to low oxygen during flight. We suggest that among different insect locomotory activities, flight performance deteriorates slowly with age, which is surprising, given that insect flight is one of the most energy-demanding activities in animals. Apparently, the superior capacity of insects to rapidly deliver oxygen to flight muscles remains little altered by ageing, but we showed that insects can become oxygen limited in habitats with a poor oxygen supply (e.g., those at high elevations) during highly oxygen-demanding activities such as flight.

## 1. Introduction

Active flight evolved independently at least four times in the history of life on Earth, leading to the origin of flying insects, pterosaurs, birds and bats [1]. Flight requires many specialized physiological and morphological adaptations [1,2] and is one of the most energy- and oxygen-demanding activities in animals [3]. For an insect, the transition from rest to flight can involve a sudden 50–100-fold increase in oxygen consumption [4] because of rapid oxygen delivery to flight muscles via a network of internal tubes. This so-called tracheal gas-exchange system appears to have evolved independently multiple times within terrestrial arthropods [5], and owing to its diffusive and convective nature and the rich oxygen supply in the air, the performance of terrestrial insects may not always be considered oxygen-limited [6,7,8,9]. Indeed, although oxygen limitation has been considered to be involved in shaping organismal traits, including preferred temperatures, physiological heat tolerance, maximal physical activity, egg laying, or even the thermal sensitivity of life-history traits [10,11,12,13,14,15,16], this phenomenon is less studied in terrestrial insects [9,17,18,19]. Nevertheless, insects are often residents of soil and litter microenvironments with hypoxic conditions, and they can disperse over long distances or occupy habitats located at different elevations and thus characterised by low oxygen partial pressure [20,21,22]. In the life cycles of many insect species, such as *Drosophila* fruit flies, different life stages occupy environments that dramatically differ in oxygen conditions [23]. Additionally, some insects are known to migrate between high- and low-elevation habitats (e.g., *Pantala flavescens* dragonflies from India to East Africa and monarch butterflies from the northeastern USA and southeastern Canada to sites in the mountains of central Mexico), and some long-distance-migrating insects take advantage of high-altitude winds [24]. Oxygen level also varies on a geological time scale and, in fact, has been invoked as one of the selective drivers of evolutionary changes in insect body size, e.g., insect gigantism in the past [5,25].

Terrestrial insects have been commonly used to study different aspects of organismal performance [20,26,27]. Such studies have aimed to understand the deterioration of performance with age [28,29]. For example, ageing *Drosophila* flies become handicapped by decreased survival rates, fertility, locomotory capacity, cardiac function or olfaction [30,31,32,33,34,35]. Importantly, ageing insects have become a model study system for addressing human ageing and searching for new methods to reduce age-related mortality in human populations [36,37]. The effects of ageing appear to be less studied for insect flight [38], but not surprisingly, emerging evidence shows that different flight characteristics undergo changes with insect age. For example, in the plum curculio *Conotrachelus nenuphar*, the total travelled distance, flight time and maximum uninterrupted flight time decrease with age [39]. In the honey bee *Apis mellifera*, the maximal wing-beat frequency and maximal average angular velocity of foragers change with age, being the lowest in precocious foragers, reaching a plateau in middle-aged foragers and decreasing in older foragers [40]. For the fruit fly *Drosophila melanogaster*, the evidence is more complex. On the one hand, the frequency of flight initiation decreases with age [41], but on the other hand, flight duration is shorter in young and old flies than in middle-aged flies [42]. Moreover, Miller et al. [43] showed that the wing-beat frequency of *D. melanogaster* may not display clear ageing effects for a considerable part of a fly’s life, followed by a sudden collapse in the oldest flies. Interestingly, ageing effects on the flight performance of *D. melanogaster* appear to depend on prior flight experience. Flies that were not allowed to fly over the course of their life lost their flight capacity faster than those that were forced to fly, with flies allowed but not forced to fly showing the latest flight capacity senescence [44]. Addressing the effect of ageing on the flight performance of insects, we measured the maximal wing-beat frequency in ageing *D. melanogaster* flies exposed to either normoxic or hypoxic conditions during flight. According to Coquin et al. [45], following temporal stress caused by severe hypoxia, older *D. melanogaster* showed slower recovery in whole-body activity, heart rate and ATP levels, suggesting that ageing flies can exhibit increased sensitivity of metabolic performance to hypoxic conditions. Accordingly, we predicted that ageing flies in our experiment would show a decreasing wing-beat frequency, especially when exposed to a challenge of reduced availability of oxygen in the air. Importantly, much of the age-dependent mortality in humans seems to be related to increasing-with-age sensitivity to ischaemic events in tissue and organs [46,47]. Therefore, the expected high tolerance of hypoxia in insects can help us identify mechanisms responsible for the vulnerability of humans to ischaemic conditions [48]. To relate our results to other potential signs of ageing, we performed another experiment on the same isolines of flies, measuring changes in the climbing capacity of flies with age. Following earlier studies of the climbing performance of *D. melanogaster* [30,49,50], we expected to observe a decreasing capacity of older flies to climb vertical walls. We generally expected that both types of locomotory activities, flight and climbing, would show deterioration with age, but because insect flight is much more metabolically challenging than climbing, we expected that flight performance would start to deteriorate earlier with age.

## 2. Materials and Methods

### 2.1. Flies

As a source of flies for this study, we used stock of *D. melanogaster* isolines maintained at the Institute of Environmental Sciences, Jagiellonian University, Krakow, Poland. The stock flies and flies used in this study were kept under controlled conditions in thermal cabinets (POL-EKO APARATURA, Wodzislaw Slaski, Poland) set to 20.5 °C and a 12 h:12 h L:D photoperiod. Containers with water placed in the cabinets provided stable humidity conditions (approximately 70%). The stock flies were kept in 40-mL vials (2.5-cm diameter, 9.5-cm height; polyurethane foam plugs) with 10 mL of cornmeal yeast food (Bloomington Drosophila Stock Center, Bloomington, IN, USA). To obtain new generations without overlap, every three weeks, fly samples from each isoline in the stock were placed in new vials with fresh food for egg laying for five days.

For the purpose of this study, we used *D. melanogaster* males originating from three isolines. To obtain an adequate number of individuals for measurements, we first performed transfers of each isoline that boosted the number of vials with flies in each isoline and created new generations that underwent development under controlled density conditions (boosting procedure). Upon each density-controlled transfer, 10 females and 10 males were placed for 48 h in large vials (68-mL vials with 20 mL of cornmeal yeast food) to obtain eggs and thus the new generation of flies. For logistical reasons, we performed two different boosting procedures, one aimed at flight performance and one aimed at climbing performance. Note that flight performance was measured in the second-generation males and climbing performance was measured in the third-generation males. To obtain flies of known age (number of days, counting from adult emergence) for each measurement, we checked vials with pupae every day to collect emerging flies with an exhauster (no anaesthesia) and transferred them to new vials with food (next steps described in detail for each performance measurement).

### 2.2. Flight Performance

Flies collected for studying flight performance were initially kept in mixed-sex groups for 2 days, when they could mate freely. Next, the flies were sexed, and males were added to new vials (68 mL) in groups of 45 individuals per vial, serving as the source of animals for the wing-beat measurements. To prevent deterioration of living conditions, the flies awaiting measurements were transferred every 10 days to new vials with fresh food. All flies awaiting measurements were kept under stock conditions.

**Figure 1:**
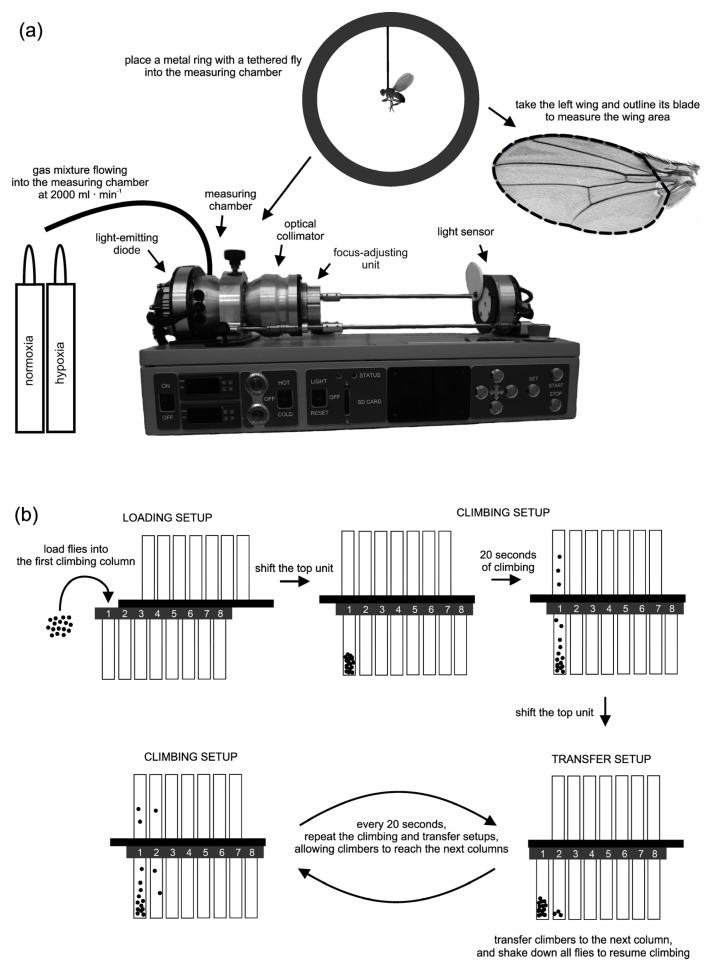
Two types of performance were measured in ageing *Drosophila melanogaster*. Flight performance was measured with an optical frequency counter (**a**). A tethered fly was mounted in an aluminium ring, which was placed in the measuring chamber. The light beam emitted by a diode travelled through the chamber, focused by an optical collimator on the sensor. The chamber was supplied with a constant flow of either a normoxic or hypoxic gas mixture. The temperature of the inflowing gas was recorded by a thermocouple placed next to the tethered fly. After the measurement, the left wing of the fly was outlined (indicated by a dotted line visible in the wing image placed in the figure) to measure the wing blade area and calculate the wing load. Climbing performance was measured on an apparatus (**b**) with 8 climbing columns as the proportion of flies loaded onto the first column that managed to leave this column by climbing onto the next columns during a series of column shifting cycles (which provided flies with a 140-s time interval that could be utilized to climb up the columns). Every 20 s, the top unit of the apparatus was shifted sideways to transfer climbers to the next columns (transfer setup). Then, the apparatus was shaken to force the climbers down, and the top unit was shifted back to the initial position to allow the flies to resume climbing (climbing setup).

Flight performance was measured as the wing-beat frequency (Hz) of tethered flies. The measurements were performed under controlled thermal and oxygen conditions (one common temperature and either normoxia or hypoxia). The measurements started with 5-day-old flies and then continued every five days until the flies had aged 50 days. Each time, 2 males per isoline were measured in each oxygen condition, with individual flies involved in the measurements only once in their lifetime (120 flies in total, representing 10 age classes, 2 oxygen conditions and 3 isolines). To measure wing-beat frequency, we used an optical frequency counter (Figure 1a) designed by Prodromus (Krakow, Poland), which in principle collects a similar type of data as the tachometers used in earlier studies [51].

The apparatus consists of a small measuring chamber made of thick aluminium with foam coating, which allowed the creation and control of thermal and oxygen conditions during measurements. A tethered fly was mounted in the centre of the chamber in a light beam travelling through the chamber to a sensor recording high-frequency light disturbances caused by wing movements (recorded 20 times per second). The light was emitted by a diode on one side of the chamber, and it was collected and focused on the sensor by an optical collimator on the other side of the chamber. The position of the collimator was adjustable, which helped create a sharp image of the fly on the sensor. The measuring chamber was connected to a gas cylinder with either a normoxic (21% O_2_) or hypoxic (10% O_2_) gas mixture (Gaz Centrum, Krakow, Poland). To generate a given oxygen condition inside the chamber, valves of a cylinder with the required gas mixture were opened, and the gas mixture was provided to the chamber at a constant rate of 2000 mL per minute with the help of a flowmeter. Before entering the measuring chamber, the gas mixture was passed through a humidifier, which provided stable humidity conditions (40%) during measurements. The temperature inside the chamber was controlled by placing the apparatus in a climatic room set to 20 °C. Two hours before each measurement session, the apparatus was switched on, and the light was set to its full intensity (manually regulated by a potentiometer). Based on our preliminary tests, this priming procedure allowed the apparatus to reach a heat-exchange equilibrium state with the ambient environment, providing stable thermal conditions during the measurements. The temperature of the inflowing gas mixtures was directly recorded to the nearest 0.05 °C by a fast-response thermocouple (0.5-mm diameter) connected to a temperature recorder (Delta OHM, Padova, Italy) placed inside the measuring chamber near a tethered fly. We aimed to expose flies to 27 °C during measurements, and the recorded temperature was on average equal to 26.4 °C (SD = 0.43). Immediately before each measurement, we briefly cold-anaesthetized each fly on ice and used UV glue to attach a thin entomological pin to the upper part of a fly’s thorax. The tethered fly was then mounted in the centre of an aluminium ring, which was then placed in a ring slot of the measuring chamber (Figure 1a). Before placing the fly into the measuring chamber, the light beam in the chamber was dimmed, and just before the recording, it was upregulated to its maximum intensity. Each recording lasted 2 min. The first recorded 15 s were discarded (habituation). The remaining recordings were analysed with a Visual Basic macro in Excel (Microsoft, Redmond, WA, USA) to find the ten-second-long interval of flight with the highest mean wing-beat frequency. We considered this value as a measure of the maximum flight performance of a fly and used it in our further analyses.

After wing-beat frequency was measured, each fly was freeze-stored to measure thorax length (mm) and wing blade area (mm^2^) and ultimately to calculate wing load as thorax length^3^·wing area^−1^ [52,53]. For each fly, we used microsurgery forceps to cut the left wing, and then a fly was placed on its right side. With the help of an ocular scale in a stereomicroscope (Olympus Corporation, Tokyo, Japan), we measured the distance from the thoracic neck edge to the tip of the scutellum to the nearest 0.02 mm (thorax length). The removed wing was mounted on a microscopic slide using ST Ultra and CV Ultra mounting media (Leica Biosystems, Nussloch, Germany). With a stereomicroscope (Nikon Corporation, Tokyo, Japan) equipped with a camera (OPTA-TECH, Warsaw, Poland) connected to a computer with OPTA View software, we took images of the wing blades. The wing area was measured by outlining the entire wing (Figure 1a) from the costal cell to the alula using ImageJ software with a LiveWire plugin (National Institutes of Health, Bethesda, MD, USA).

### 2.3. Climbing Performance

Flies collected for studying climbing performance were sexed immediately upon collection, and males were added to new vials (40 mL), serving as the source of animals for the measurements. For each isoline, we maintained three vials with 15 males per vial (total of 45 males per isoline). To prevent deterioration of living conditions, flies awaiting measurements were transferred every 7 days to new vials with fresh food. All flies awaiting measurements were kept under stock conditions.

Climbing performance measurements were performed at room temperature (23 °C) with no manipulation of oxygen conditions (normoxic ambient air). Before each measurement session, vials with flies were exposed to room conditions for 10 min to allow the flies to habituate to the measurement conditions. The measurements started on the 7-day-old flies and then continued every seven days (for logistical reasons, in one case, the flies were tested after an 8-day interval) until the flies had aged to 43 days. For each measurement session, flies from all three vials were pooled within each isoline and placed together in our testing apparatus. After the measurement, the flies were randomly divided back into three equally large groups (per isoline), and each group was placed in a new vial with fresh food, awaiting the next measurement session in the following week. Our climbing assays were performed with the help of a modified version of the apparatus designed by [54], which utilized the tendency among flies to climb up vertical walls, called negative geotaxis [30]. In principle, our apparatus (Figure 1b) consisted of a set of climbing columns made of transparent test tubes vertically mounted in a Plexiglas frame, and it had two movable parts (top and bottom) to shift climbing flies from one column to the next column in the row. To start a climbing assay, flies were loaded into the first column and allowed to climb up the column for 20 s. The successful climbers were then transferred to the next column by shifting the top unit of the apparatus and shaking off flies to the bottom of the columns (Figure 1b). After shifting the top unit back to its initial position, the climbing assay was resumed. The shifting/climbing cycles (7 in total) were repeated every 20 s, which allowed climbing flies to enter the next columns in the row until the fastest climbers reached the last (8th) column. Climbing performance was measured as the proportion of flies that managed to climb out of the first column during the whole measurement session (hereafter, the proportion of climbers), which lasted 140 s in total.

### 2.4. Statistical Analysis

Statistical analysis was performed in R 4.0.3 software [55] with the lme4 [56], car [57], ggplot2 [58] and emmeans [59] packages. We used a general linear mixed model (GLMM) with a Wald F test to fit models to our data and assess the statistical significance of model components. The GLMM for flight performance included isoline as a random factor and age and oxygen condition as two fixed grouping factors. The model also included wing load as a numeric covariate and the interaction between oxygen condition and age. The GLMM for climbing performance included isoline as a random factor and age as a fixed grouping factor. To meet the assumptions of the parametric tests, prior to the analysis of the proportion of climbers, we applied arcsine square root transformation to our proportion data [60].

## 3. Results

**Figure 2:**
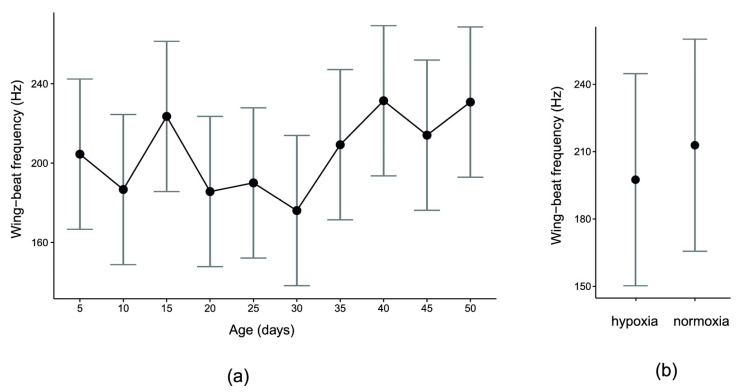
Males of *Drosophila melanogaster* showed significant differences in flight performance among age classes, but these changes did not show any consistent age pattern (**a**). Flies achieved lower flight performance in hypoxic (10% O_2_) than in normoxic (21% O_2_) conditions (**b**). Flight performance was measured as the maximum wing-beat frequency of tethered flies (see Figure 1a for technical measurement details). The means (95% CIs) were estimated with a general linear mixed model.

The GLMM for flight performance showed that wing-beat frequency changed significantly among age classes (F_9, 97.106_ = 3.36, *p* < 0.002), but these changes did not show any consistent age pattern (Figure 2a), suggesting that ageing flies did not decrease or systematically increase their flight performance. The model also showed that wing-beat frequency was lower in hypoxic than in normoxic conditions (Figure 2b, F_1, 97.155_ = 4.84, *p* = 0.03).

**Figure 3:**
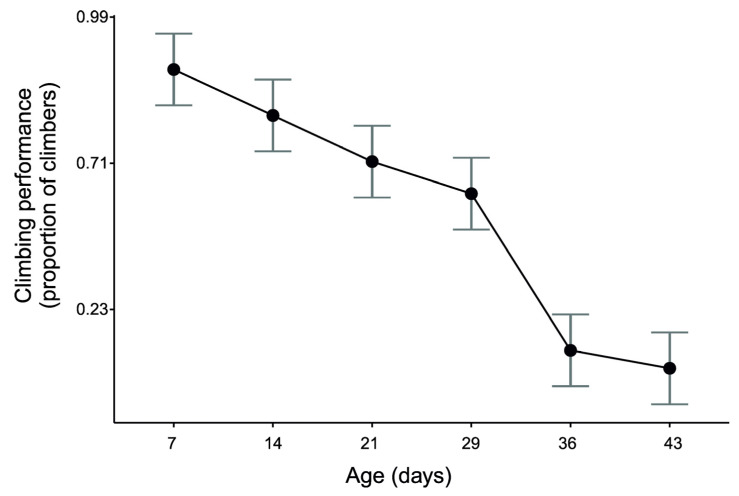
Males of *Drosophila melanogaster* showed systematic deterioration of climbing performance with age. Climbing performance was measured by calculating the proportion of flies that climbed out of a vertical column during a 140-s climbing assay (see Figure 1b for technical measurement details). The means (95% CIs) were estimated with a general linear mixed model performed on transformed data. For convenience, values on the vertical axis are shown as proportion units after inverse transformation.

The effects of wing load and the interaction between oxygen condition and age were not significant (F_1, 87.002_ = 0.08, *p* = 0.78 and F_9, 97.175_ = 1.23, *p* = 0.28, respectively). The nonsignificant interaction indicates that the effects of oxygen conditions on wing-beat frequency were consistent among the age classes. The GLMM for climbing performance showed that the proportion of climbers systematically decreased in consecutive age classes (Figure 3, F_5, 10_ = 56.38, *p* < 0.00001).

## 4. Discussion

We monitored two types of locomotor performance in ageing males of *D. melanogaster*, which at the end of our study approached an age of either 43 days (climbing assays) or 50 days (flight assays) after eclosion. Given the expected strong thermal dependence of *D. melanogaster* longevity [61], we considered 40- to 50-day-old flies originating from our thermal regime (20.5 °C) to have already hit the adult mid-lifespan. In addition to developmental temperatures [62], the rate at which flies approach this life stage can also depend on prior flight experience [43] and genetic characteristics of the flies, such as the level of inbreeding [63] or source population [61]. We observed clear signs of physiological senescence appearing over the studied flies’ age span, but interestingly, the two studied locomotor activities (climbing and flight) followed inconsistent age patterns. In line with predictions, the climbing capacity steadily decreased with age, with the oldest flies (43 days) characterized by nearly 90% lower climbing activity than the youngest flies (7 days). This pattern agrees with the findings of earlier studies, which used the geotaxic behaviour of flies to demonstrate the decreasing capacity of ageing flies to climb vertical walls [30,49]. In contrast, while the maximum wing-beat frequency of tethered flies varied significantly among the different age groups (from 5- to 50-day-old flies), we did not find any consistent age dependence in this variance. Against our predictions, this finding suggests that the flight performance of *D. melanogaster* does not decrease systematically with fly age. This result is surprising given the evidence that ageing *D. melanogaster* undergoes deterioration in cardiac performance [64] as well as in an array of other types of performance, including negative geotaxis, exploratory activity, fast phototaxis, reproduction, sperm competition, innate immunity, circadian rhythmicity, noncircadian rest, flight duration, learning ability, olfactory memory, and even stress resistance (summarised by Gargano et al. [30]). Similarly, studies of other insect species provide evidence for the rapid deterioration of organismal performance with age. For example, males of *Gryllus campestris* crickets decrease dominance in fights and calling effort with age [65]. Spontaneous locomotion and walking have been shown to decline with age in the *Blaberus discoidalis* cockroach [66], and older *Apis mellifera* bees are characterized by reduced foraging success [67]. Nevertheless, our flight performance results support the evidence of Miller et al. [43] and Petrosyan et al. [68], showing that the wing-beat frequency of *D. melanogaster* can remain unaltered for a considerable part of its initial life, rapidly deteriorating only among very old flies. It is likely that extending our flight assays to include much older flies would result in recording wing beats with decreased frequency. Note, however, that we continued flight assays as long as we had access to flies, which decreased in number over time due to natural mortality and random losses caused by transfers and handling. We also observed (but did not quantify) that the oldest age classes in our study accumulated individuals with physically damaged wings, and such specimens were excluded from the measurements. Altogether, we conclude that evidence for ageing insects indicates that organismal functions decline at different rates with age, and given our results, it appears that the capacity to move wings at high frequencies declines much more slowly than other locomotor activities. For a long time, *D. melanogaster* was believed to be a poor disperser, but field experiments demonstrated that flies can cover an average of 150 metres [69], with the longest confirmed daily distances reaching 7–10.2 km [70,71]. Therefore, it would be interesting to know more about how the capacity to achieve high flight speeds corresponds to dispersal and fitness in natural populations, but we are only beginning to collect information on the biology of wild *Drosophila* flies [72,73,74]. Nevertheless, flight capacity is a crucial and integral component of insects’ life histories that shapes dispersal patterns in wild populations and undergoes evolution driven by the local availability of resources such as food, mates and oviposition sites [75,76,77,78]. Therefore, the maintenance of flight muscle performance with age would likely be prioritized by natural selection over the maintenance of the performance of muscles involved in walking. Nevertheless, given the short expected lifespan in wild fruit flies of ca. 14 days [69] and the rapid decline in oviposition rates with fly age [79], it seems unlikely that the extended maintenance of flight performance, beyond the life stage with high expected egg-laying activity, would be promoted by natural selection in flies. Interestingly, Petrosyan et al. [68] showed that *D. melanogaster* mutants characterised by increased longevity had a higher wing-beat frequency than control flies, but this effect disappeared in older flies, when wing-beat frequency declined in both groups and all flies ultimately expressed similar flight characteristics. Certainly, wing-beat frequency is not the only flight characteristic relevant to discussions of the flight performance of ageing insects. For example, evidence shows that ageing flies can reduce the duration of flight or initiate flight less often [38,41,42], indicative of declining flight activity with age. All things considered, at this point, we remain far from understanding the precise physiological mechanisms that determine age-related changes in the locomotory performance of insects, especially the relative contribution of neural and muscular processes. Nevertheless, insect leg movements are powered by muscles with synchronous contractions, while wing movements, at least in all flies and many other small insect species, are powered by muscles functioning with asynchronous contractions [80,81,82]. In the synchronous muscles, contractions are directly stimulated by impulses arriving from motor nerves, with one impulse per contraction. Relaxation of the muscle, and thus its recovery to an activation state, requires molecular work that restores the Ca^2+^ potential on the sarcoplasmic reticulum, but this imposes a limit on the maximal frequency of contractions (~100 Hz). In contrast, asynchronous indirect flight muscles can undergo contractions at much higher frequencies (up to 10 times) without the need to restore the Ca^2+^ potential and stimulate the muscles by neurons after each contraction. All thanks to the self-perpetuating activation/deactivation cycles, which are performed by two antagonistic muscle groups in the thorax—a contraction of one muscle group enforces a relaxation of the other muscle group, and vice versa. In effect, the contraction frequency of the synchronous leg muscles is directly linked to the frequency of neural impulses (1:1), whereas the contraction frequency of the asynchronous indirect flight muscles greatly depends on the resonance and deformation properties of the thoracic exoskeleton, with the much less intense involvement of neural stimulation, which plays a regulatory role [81,83]. Fragmentary evidence suggests that the ageing flies of *D. melanogaster* accumulate molecular damage in the leg and flight muscles [84], but it remains to be studied whether this accumulation occurs at similar rates in the two muscle types. It would also be fascinating to explore whether the contrasting modes of contraction stimulation in the synchronous and asynchronous muscles can make the performance of leg muscles more prone to the age-related decline in neural functions.

Insect flight muscles are completely aerobic [82], indicating the central importance of sustainable oxygen supply to mitochondria in the flight performance of insects. When performing highly ATP-demanding activities, such as active flight, under acute hypoxia, insects can accumulate excessive numbers of protons (acidosis) and calcium ions in muscle tissue, which can lead to molecular damage to contractile structures and mitochondria [45,85,86,87]. Evidence from Coquin et al. [45] suggests that ageing *D. melanogaster* can become increasingly more vulnerable to this hypoxia-driven damage, but we did not find evidence that flight performance was becoming more hypoxia sensitive in older flies. Apparently, the hypoxic conditions used in our flight assays were challenging because hypoxia-exposed flies (10% O_2_ in the air) flapped their wings at lower frequencies than normoxia-exposed flies (21% O_2_ in the air). Nevertheless, our normoxia- and hypoxia-exposed flies maintained their wing-beat frequency with age in similar manners, indicating that a reduced supply of air oxygen equally impeded the physiological work of flight muscles in young and old flies. Although we did not find evidence of ageing effects on hypoxia tolerance, the oxygen limitation of flight performance is an important result of our study because this phenomenon has only occasionally been studied in insects, and available evidence seems to be inconsistent. For example, in support of our results, Shiehzadegan et al. [17] showed that *D. melanogaster* flies raised under normoxia were more likely to fly under normoxia than under hypoxia and hyperoxia at 25 °C. Joos et al. [88] demonstrated a decreased wing-beat frequency in bees exposed to air with low oxygen partial pressure. In contrast, Chadwick and Williams [89] studied two *Drosophila* species, close relatives of the species (*D. melanogaster*) studied here, and demonstrated that wing-beat frequency increased with either low barometric air pressure or low air density, but these effects were not linked to differences in oxygen partial pressure. In fact, neither hypoxia nor hyperoxia changed wing-beat frequency in the studied *Drosophila*. Moreover, in agreement with our results, Lighton and Schilman [90] demonstrated that the upper critical thermal maxima of *D. melanogaster* are lowered by hypoxia, but Klok, Sinclair, and Chown [9] did not find such effects for the *Gonocephalum simplex* beetle. Certainly, such a complex picture can indicate two important and not mutually exclusive phenomena, which seem worth addressing explicitly in future studies. Demonstrating the oxygen limitation of insect performance can depend on which type of activity is studied [88] and which oxygen partial pressure is used with reference to the physiological adaptations of the studied insects [88,90].

## 5. Conclusions

Overall, we demonstrated that low oxygen impedes *Drosophila* flight performance, but flies maintain their flight abilities with age, and younger and older flies show similar levels of tolerance to low oxygen during flight. Apparently, the superior capacity of insects to rapidly deliver oxygen to flight muscles remains little altered by ageing, but it becomes oxygen limited when insects perform highly oxygen-demanding activities in habitats with poor oxygen supply. Taking a wider evolutionary and ecological perspective, our results shed light on the challenges to flying insects imposed by the supply of environmental oxygen, e.g., changes on a geological scale [5,25] or along an elevation gradient [91,92,93]. For example, *Apis mellifera* honeybees have been recorded at elevations up to 3000 m but struggle to sustain flight capacity above that level [88]. Interestingly, alpine bumble bees were recorded above 5600 m, and they were shown to have the ability to maintain hovering flight under barometric pressures that occur above 9000-m elevations [92]. Analysis of latitudinal and seasonal variation in the partial density of oxygen in the air suggests that insects can also experience varying oxygen conditions on geographic and temporal timescales [94], but the biological significance of this type of variance needs to be studied. It is important to note here that prolonged exposure to hypoxia can affect insects in different ways than the acute hypoxia studied here, changing profiles of gene expression and protein production, the efficiency of ATP production, growth rates, development, the architecture of the tracheal system, cell size, and body size, among other features [48,95]. Perhaps many of these developmental responses represent adaptive responses driven by a changing balance between oxygen supply and metabolic demand. Importantly, adaptive responses to this balance are predicted to at least partly explain developmental responses of ectotherms to warmer conditions [14,96,97,98], such as earlier maturation and smaller adult size, called the temperature-size rule [99,100], and/or developing organs built from smaller cells [101,102]. We are not aware of any studies on insects that explicitly compared the fitness effects of these responses among environments differing in metabolic demand and oxygen supply. Nevertheless, experiments on a freshwater rotifer demonstrated that small phenotypes built of small cells became more fertile than large phenotypes built of large cells when conditions in the environment became warm (increased oxygen demand) and hypoxic (decreased oxygen supply) [13]. We believe that oxygen limitation should be more frequently addressed in future studies aimed at understanding the origin of insects’ life strategies.

## Data Availability

Available in the Supplementary Materials.

## Supplementary Materials

The following are available online at https://www.mdpi.com/article/10.3390/biology10040327/s1.
